# The Average IFN-**γ** Secreting Capacity of Specific CD8^+^ T Cells Is Compromised While Increasing Copies of a Single T Cell Epitope Encoded by DNA Vaccine

**DOI:** 10.1155/2012/478052

**Published:** 2012-11-01

**Authors:** Yanmin Wan, Jing Wang, Haizhu Zhou, Zhidong Hu, Xiaonan Ren, Jianqing Xu

**Affiliations:** ^1^Shanghai Public Health Clinical Center, Fudan University, Shanghai 201508, China; ^2^Institutes of Biomedical Sciences, Fudan University, Shanghai 200032, China; ^3^Key Laboratory of Medical Molecular Virology of Ministry of Education/Health, Fudan University, Shanghai 200032, China; ^4^School of Biology and Basic Medical Sciences, Soochow University, Suzhou 215123, China

## Abstract

Previous studies suggested that both the frequency and the mean fluorescence intensity (MFI) of cytokine secreting T cells could be of great value for immunogenicity evaluation of a vaccine. In this study, by constructing epitope-based DNA vaccines encoding a previously identified CD8^+^ T cell epitope, we investigated the influence of multiplying epitope copies on both the frequency and the MFI of specific IFN-**γ** secreting CD8^+^ T cells. We found that frequencies of specific CD8^+^ T cell could be improved by multiplying epitope copies, while the MFI of IFN-**γ** secreted by epitope-specific CD8^+^ T cells decreased synchronously. And further analysis showed that the decrease of MFI was not caused by the functional avidity variation of CD8^+^ T cell receptor.

## 1. Introduction

Traditional vaccines have dramatically diminished morbidity and mortality of a large number of infectious diseases, while their success cannot be easily translated into developing of vaccines against HIV, malaria, and cancer [1]. Novel approaches are urgently needed. In formality of either recombinant vectored vaccines or synthetic peptides, epitope-based vaccine represents one of these emerging approaches. Taking benefits of well-defined epitopes with a minimal structure influence, this epitope-based approach can focus immune responses on conserved epitopes and also increase the potency and breadth of specific immune responses [2, 3]. 

Although it has been widely employed in vaccine development against HIV, HCV, HBV, HPV, cancer, and Helicobacter pylori [4, 5], its relatively weak immunogenicity still remains a major restraint for the practical application of epitope-based vaccines [6]. Previous studies suggested that the immunogenicity of epitope-based DNA vaccines could be enhanced by introducing intracellular targeting signals to direct the encoded gene product to the endoplasmic reticulum (ER) [7, 8], by including Pan HLA-DR epitope (PADRE) to support the development of specific immune responses [7, 9] and by incorporating spacer sequences between epitopes to optimize epitope processing [10]. Most recently, another study also suggested that increasing the copy number of epitope coding gene could augment the magnitude of specific T cell responses against a carcinoembryonic-antigen-(CEA-) derived epitope [11]. However, in most of these studies, the frequency of specific cytokine (IFN-*γ*) secreting T cells was typically used as the parameter to measure the specific T cell responses. But this might not be the best indicator of protective T cell responses, because several lines of evidence have already suggested that the mean fluorescent intensity (MFI) also was an important coparameter [12–14]. In order to further improve the immunogenicity of epitope-based DNA vaccine, in this study, we constructed epitope-based DNA vaccines by using a combined immunogenicity-enhancing design. And its influences on both the frequency and the MFI of specific IFN-*γ* secreting T cells were evaluated in mice. Surprisingly, we found that the average IFN-*γ* secreting capacity of specific CD8^+^ T cells was compromised while increasing the copy number of epitope encoding sequence in DNA vaccine.

## 2. Material and Methods 

### 2.1. Epitope-Based DNA Vaccine Design and Construction

**Figure 1:**
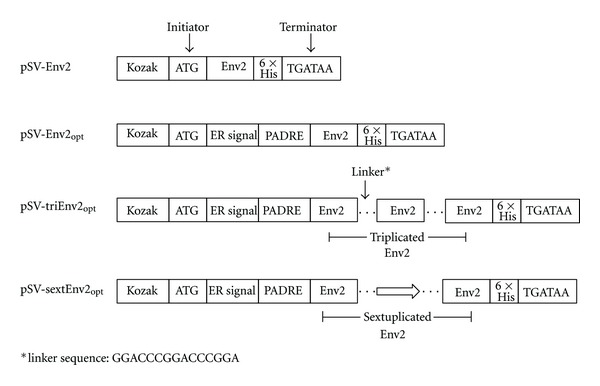
The diagram of epitope-based DNA vaccine design. To construct pSV-Env2_opt_, pSV-triEnv2_opt_, and pSV-sextEnv2_opt_, a Kozak sequence, an ER signal sequence, and a universal Th2 epitope (Pan DR epitope, PADRE) were introduced into the 5′ end of each epitope encoding gene and a 6 × His-tag was added at the 3′ end. A linker sequence was also inserted between adjacent epitopes. pSV-Env2 was constructed without adding ER signal sequence nor PADRE.

A previously identified CD8^+^ T cell epitope derived from HIV-1_RL42_ Env (GIRKNYQHLWRWGTM, designated as Env2 [15]) was used for epitope-based DNA vaccine construction. Mini-genes encoding single, triplicate, or sextuplicate copies of this epitope were synthesized and inserted into plasmid vector pSV1.0 as DNA vaccines. To enhance their expression efficiency and immunogenicity, a Kozak sequence, an ER signal sequence, and a universal Th2 epitope (Pan DR epitope, PADRE) were introduced into the 5′ end of the epitope coding genes and a 6 × His-tag was added at the 3′ end for detecting their expression readily (Figure 1). A previously reported linker [10] was inserted between adjacent repeated epitopes to facilitate epitope processing. Moreover, a single copy of Env2 without adding ER signal and PADRE sequences was constructed as nonoptimized control, designated as pSV-env2. All DNA vaccines used for immunization were prepared by using an Endofree Plasmid Giga Kit (Qiagen, no. 12391).

### 2.2. *In Vitro *Expression Assay

HEK 293T cells plated in 6-well plates were transfected with 4 *μ*g of each DNA vaccine by using Turbofect in vitro transfection reagent (Fermentas, no. R0531) according to the manufacturer's instructions. Briefly, 48 hours after transfection, cells were harvested and lysed with modified RIPA lysis buffer (Pierce, no. 87788) on ice and centrifuged at 12,000 g for 5 mins. The supernatant was then incubated with mouse anti-His mAb (Beijing Zhongshan Biotech, no. TA-02) overnight at 4°C. And then, antigen-antibody complex was pulled down by using Protein A/G Agarose Beads Kit (Beyotime, no. P2012). During western-blotting assay, the mouse anti-His mAb (Beijing Zhongshan Biotech, no. TA-02) was used as the first antibody and HRP-linked goat anti-mouse IgG (Beijing Zhongshan Biotech, no. ZB-2305) was used as the second antibody. 

### 2.3. Animals and Vaccination

 All animal experiments were reviewed and approved by the Institutional Animal Care and Use Committee (IACUC) of Shanghai Public Health Clinical Center and were performed in accordance with relevant guidelines and regulations in China. 28 six-week-old female C57BL mice were randomly divided into 4 groups (7 mice for each). 100 *μ*g of purified plasmid DNA dissolved in 100 *μ*L sterile PBS was inoculated intramuscularly into *tibialis anterior* for three times at weeks 0, 2, 4, and all mice were sacrificed 2 weeks after the final vaccination (schedule shown in Table 1). Splenocytes were freshly collected for intracellular cytokine staining (ICS) and IFN-*γ* Elispot assay.

### 2.4. Peptide Stimulation and Intracellular Cytokine Staining Assay

Fresh isolated mice splenocytes were adjusted to the concentration of 2 × 10^7^ cells/mL and plated into round bottom 96-well cell culture plate at 50 *μ*L/well (1 × 10^6^ cells per well) with addition of 50 *μ*L Env 2 peptide (GIRKNYQHLWRWGTM, kindly provided by NIH AIDS Research & Reference Reagent Program); the final concentration is 5 *μ*g/mL. After one hour incubation at 37°C with 5% CO_2_, Golgi blocking reagents, Brefeldin A (eBioscience, no. 00-4506-51), and Monesin (eBioscience, no. 00-4505-51) were added into each well at the final concentrations of 1 ug/mL and 1 uM, respectively. Then, the plates were incubated again at 37°C with 5% CO_2_ for another 5 hours. After incubation, the splenocytes were first stained with anti-mouse CD3 mAb (PerCP, BD Pharmingen), anti-mouse CD4 mAb (Pacific Blue, BD Pharmingen), and anti-mouse CD8 mAb (PE, BD Pharmingen) at 4°C for 30 minutes. Then, the cells were fixed and permed with fix/perm buffer (BD bioscience, no. 554715). After washing, anti-mouse IFN-*γ* mAb (FITC, BD Pharmingen) and anti-mouse IL-2 mAb (APC-Cy7, BD Pharmingen) were added to each well and incubated at 4°C for another 30 minutes. Finally, the cells were washed and analyzed with BD FACS Aria I. The data were analyzed with flowjo7.6.1(Tree Star, Inc).

### 2.5. T Cell Functional Avidity Assay

A previously reported IFN-*γ* ELSPOT-based method was employed in T cell functional avidity assay [16]. Briefly, mice splenocytes were adjusted to the concentration of 4 × 10^6^ cells/mL and plated into a precoated 96-well ELISPOT plate (BD Bioscience, no. 551083) at 50 *μ*L/well (2 × 10^5^ cells per well) with addition of 50 *μ*L Env2 peptide. The final concentration of Env2 peptide ranged from 5 ug/mL to 0.000064 ug/mL with 5-fold serial dilution. ELISPOT plates were incubated for 20 hours. After incubation at 37°C with 5% CO_2_ for 20 hours, the ELISPOT plates were developed according to the manual and read with Immunospot Reader (Champspot III, Beijing Sage Creation Science, China). Results were expressed as spot-forming cells (SFCs) per million splenocytes.

### 2.6. Statistical Analysis

Comparisons among 3 or more groups were done by using the method of One-way ANOVA, and comparisons between two groups were done by the method of *t*-test. Significant difference was defined as *P* ≤ 0.05.

## 3. Results

### 3.1. Epitope-Based DNA Vaccines Modified by Adding Kozak, ER Signal, and PADRE Sequences Could Be Expressed Efficiently *In Vitro*

**Figure 2:**
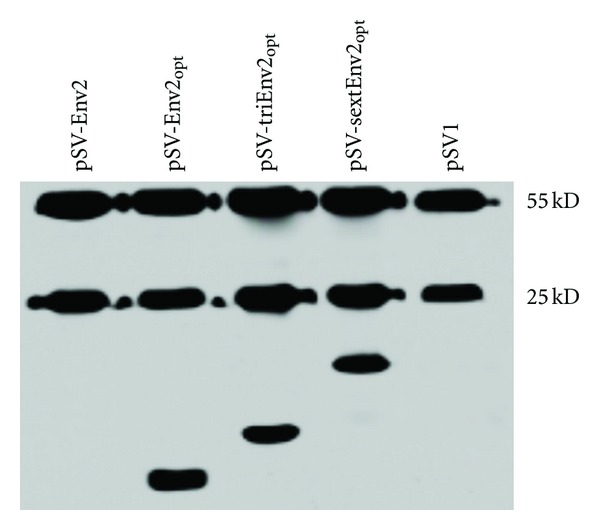
*In vitro* expression of epitope-based DNA vaccines. The expression products of epitope-based DNA vaccines were first precipitated with mouse anti-His antibody (IgG) and then detected by western-blotting. The coprecipitated heavy (55 KD) and light chains (25 KD) of mouse IgG served as internal controls. The relative expression levels were similar among pSV-Env2_opt_, pSV-triEnv2_opt_, and pSV-sextEnv2_opt_, while, the expression of pSV-Env2 could not be detected.

48 hours after transfection, HEK 293T cells were collected for immunoprecipitation and western-blotting assay. As being shown in Figure 2, all the DNA vaccines modified by adding Kozak, ER signal, and PADRE sequences could be expressed *in vitro*. And their expression efficiencies were similar, judging from the relative optical density between epitope peptide bands and their coprecipitated mouse anti-His mAb (IgG) bands. But the expression of pSV-Env2 could not be detected, which might be due to the lack of ER signal sequence.

### 3.2. Increasing Epitope Copy Number Could Significantly Augment the Frequency of Specific IFN-*γ* ^+^CD8^+^ T Cells While Lower down Their Mean Fluorescence Intensity

**Figure 3:**
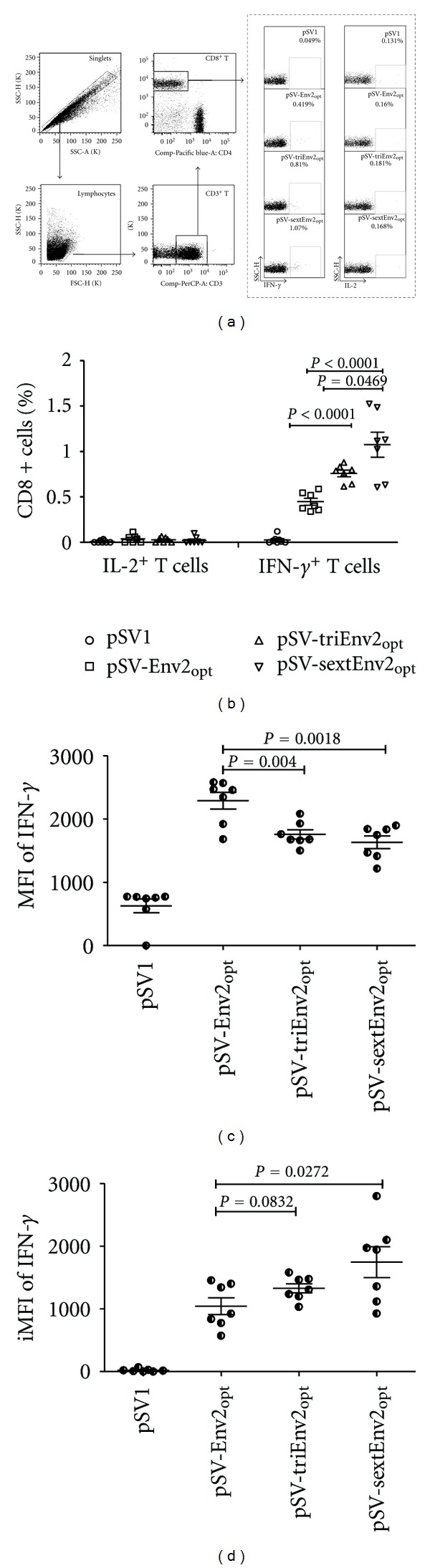
Immunogenicity comparison among groups of mice immunized with different epitope-based DNA vaccines. 100 *μ*g of purified plasmid DNA solved in 100 *μ*L sterile PBS was inoculated intramuscularly into tibialis anterior for three times at weeks 0, 2, and 4. 2 weeks after the final vaccination, the mice were sacrificed and splenocytes were freshly collected for intracellular cytokine staining (ICS) assay. (a) Representative flow cytometric analysis of IFN-*γ* and IL-2 secretion after stimulating mice splenocytes with Env2 peptide. The cytokines secretion profiles shown were gated on CD3^+^CD8^+^ T cells. (b) pSV-Env2_opt_, pSV-triEnv2_opt_, and pSV-sextEnv2_opt_ could elicit appreciable IFN-*γ* secretion in CD8^+^ T cells and the frequencies of IFN-*γ* ^+^ CD8^+^ T cells ranked as pSV-Env2_opt_ < pSV-triEnv2_opt_ < pSV-sextEnv2_opt_ (*P* < 0.0001). No significant IL-2 response was observed in any of the groups. (c) Compared with pSV-Env2_opt_(2289 ± 348.7), the mean fluorescence intensity of IFN-*γ* in IFN-*γ* ^+^CD8^+^ T cells elicited by pSV-triEnv2_opt_ (1755 ± 192.1) and pSV-sextEnv2_opt_ (1631 ± 263.7) was significantly lower. (d), the integrated mean fluorescence intensity (iMFI) of mice immunized with pSV-sextEnv2_opt_ was significantly higher than mice immunized with either pSV-triEnv2_opt_ or pSV-Env2_opt_. The iMFI was calculated as multiplying the frequency of IFN-*γ* ^+^ CD8^+^ T cells by the corresponding mean fluorescence intensity of IFN-*γ*.

As the expression of pSV-Env2 could not be detected *in vitro*, we thus excluded it from *in vivo* immunogenicity test. All the other three epitope-based DNA vaccines were included in mice immunization and the empty plasmid vector (pSV1.0) was used as mock control (Table 1). 2 weeks after the final inoculation, mice splenocytes were isolated for intracellular cytokine staining assay. Gating strategy of flow cytometric assay is illustrated in Figure 3(a). Our data showed that all three epitope-based DNA vaccines could elicit appreciable IFN-*γ* responses in CD8^+^ T cells, while no significant IL-2 secretion was observed in all groups (Figure 3(b)). One-way ANOVA analysis showed that the frequency of epitope-specific IFN-*γ* ^+^CD8^+^T cells induced by epitope-based DNA vaccines ranked as pSV-Env2_opt_[(0.45 ± 0.101)%] < pSV-triEnv2_opt_[(0.76 ± 0.097)%] < pSV-sextEnv2_opt_[(1.07 ± 0.364)%] (*P* < 0.0001) (Figure 3(b)), and this was also supported by the data of IFN-*γ* ELISPOT assay (See Figure S1 in Supplementary Material available online at doi: 10.1155/2012/478052). But, surprisingly, the MFI of IFN-*γ* in IFN-*γ* ^+^CD8^+^ T cells decreased along with the increased copy number of Env2 epitope, which peaked in pSV-Env2_opt_ group (2289 ± 348.7) and was less in pSV-triEnv2_opt_ group (1755 ± 192.1) and pSV-sextEnv2_opt_ group (1631 ± 263.7) with significant statistical difference (*P* < 0.0001) (Figure 3(c)).

Previous study suggested that integrated mean fluorescence intensity (iMFI, defined as the product of the frequency of specific T cells multiplied by their mean fluorescent intensity) for IFN-*γ*, IL-2, and TNF-*α* of mouse CD4^+^ T cells independently correlated with protection in a challenge model better than either the percentage or the MFI alone [14]. Hence, in this study, we further compared the iMFI among different groups and found that only the pSV-sextEnv2_opt_ could significantly enhance the iMFI of epitope-specific IFN-*γ* ^+^ CD8^+^ T cells (Figure 3(d)).

### 3.3. The MFI of IFN-*γ* in IFN-*γ* ^+^CD8^+^ T Cells Was Not Affected by the Functional Avidity of TCR

**Figure 4:**
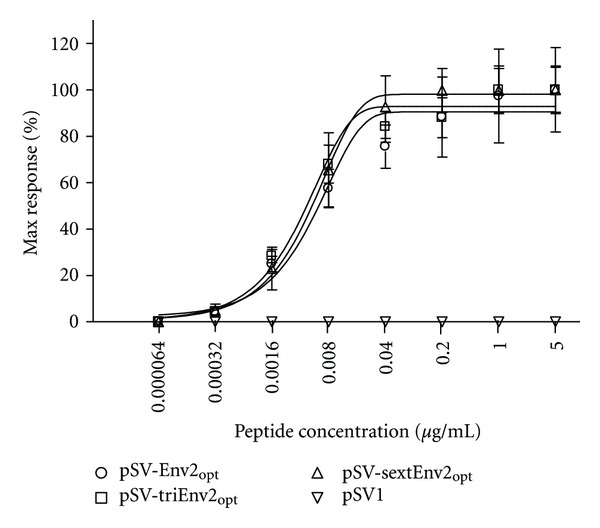
Functional TCR avidity of epitope-specific T cells was similar among all groups. Env2-specific T cell avidity was detected by using an IFN-*γ* ELISPOT. The dose-effect curve showed no significant difference among groups vaccinated with pSV-Env2_opt_, pSV-triEnv2_opt_, and pSV-sextEnv2_opt_.

To clarify whether the decreased MFI of IFN-*γ* was caused by variation of functional avidity of TCR, we did functional avidity assay by using the method of IFN-*γ* ELISPOT. As being shown in Figure 4, the functional TCR avidity of specific T cells was similar among groups immunized with pSV-Env2_opt_, pSV-triEnv2_opt_, and pSV-sextEnv2_opt_, which indicated that the TCR avidity did not affect the MFI of IFN-*γ* in IFN-*γ* ^+^CD8^+^ T cells.

## 4. Discussions

Epitope-based vaccine represents an alternative and complementary approach for vaccine development; yet, its immunogenicity needs to be improved [4]. To optimize the design of epitope-based vaccine, in this study, we constructed DNA vaccines encoding single, triple, or sextuple copies of a previously defined CD8^+^ T cell epitope [15]. 

*In vitro* expression assay showed that the expression of DNA vaccine encoding a single copy of Env2 without adding ER signal or PADRE sequence could not be detected. This might be the consequence of missing ER signal sequence, which has been suggested to be able to significantly enhance the expression of epitope-based DNA vaccine [7, 8]. But, the exact mechanisms need to be elucidated by further experiments. Since repeated WB assays consistently showed the *in vitro* expression of pSV-Env2 could not be detected, we then excluded it from mice immunization and the immunogenicity comparisons were only done among mock, pSV-Env2_opt_, pSV-triEnv2_opt_, and pSV-sextEnv2_opt_. 

Both ICS and IFN-*γ* ELISPOT assays were applied to do immunogenicity evaluation in this study. Although ICS has been intensively used in T cell response detection, most studies focused mainly on the frequency of specific T cells [17, 18], which is not the only measurement that can be accomplished by this method [1]. As being suggested by previous studies, the mean fluorescence intensity could also be of high importance for efficacy assessment of a vaccine [14]. Therefore, in this study, we took both the frequency and the MFI of epitope-specific CD8^+^ T cells into consideration. Our data showed that the frequency of epitope-specific CD8^+^ T cells could be significantly improved after increasing the epitope copy number, which was in consistent with previous reports [11]. A tendency of linear correlation between replication number of the epitope and specific CD8^+^ T cells frequency was observed (Supplementary Figure S2). 

On the contrary, the mean fluorescence intensity of IFN-*γ* in epitope-specific CD8^+^ T cells diminished significantly along with the increase of epitope copy number contained in DNA vaccine, which suggested the average capacity of IFN-*γ* secretion was compromised. As a previous study suggested that low avidity T cell displayed impaired cytokine secretion capacity [19], we thus compared the functional avidity of epitope-specific T cells by using the method of IFN-*γ* ELISPOT. Yet, no significant difference was found among groups immunized with pSV-Env2_opt_, pSV-triEnv2_opt_, and pSV-sextEnv2_opt_, which indicated the functional avidity of TCR did not affect the average amount of IFN-*γ* secretion. Few other potential mechanisms may also be involved, including the activation and differentiation status of specific CD8^+^ T cells and their TCR usage, which need to be further investigated in future. 

In spite of the unclarified mechanisms, our data clearly showed that increasing epitope copies has inversed influences on the frequency and the average IFN-*γ* secreting capacity of epitope-specific CD8^+^ T cells, which should be taken into consideration in optimizing epitope-based vaccine.

## Supplementary Material

To confirm the finding that increasing epitope copies could significantly augment the magnitude of specific T cells, 3 mice from each group were randomly selected for IFN-**γ** ^+^ ELISPOT assay. Our data showed that the frequency of IFN-**γ** ^+^ T cells could be significantly improved by increasing the epitope copies (*P* = 0.0007, one way ANOVA, supplementary figure 1). Additionally, a significant linear correlation was observed between the frequency of specific IFN-**γ** ^+^CD8^+^ T cells and the epitope copy number (supplementary figure 2).

## Figures and Tables

**Table 1:** Vaccination schedule of mice. All mice were intramuscularly inoculated with DNA vaccines.

Group	Week 0	Week 2	Week 4	Week 6
Mock (7 mice)	pSV1.0 (100 *μ*g/mice)	pSV1.0 (100 *μ*g/mice)	pSV1.0 (100 *μ*g/mice)	Sacrifice
pSV-Env2_opt_ (7 mice)	pSV-Env2_opt_ (100 *μ*g/mice)	pSV-Env2_opt_ (100 *μ*g/mice)	pSV-Env2_opt_ (100 *μ*g/mice)	
pSV-triEnv2_opt_ (7 mice)	pSV-triEnv2_opt_ (100 *μ*g/mice)	pSV-triEnv2_opt_ (100 *μ*g/mice)	pSV-triEnv2_opt_ (100 *μ*g/mice)	
pSV-sextEnv2_opt_ (7 mice)	pSV-sextEnv2_opt_ (100 *μ*g/mice)	pSV-sextEnv2_opt_ (100 *μ*g/mice)	pSV-sextEnv2_opt_ (100 *μ*g/mice)	
