# Supplementary figures and images for: The Average IFN-γ Secreting Capacity of Specific CD8+ T Cells Is Compromised While Increasing Copies of a Single T Cell Epitope Encoded by DNA Vaccine
Source: Clin Dev Immunol. 2012 Nov 1;2012:478052. doi: 10.1155/2012/478052 (PMC3509377; doi:10.1155/2012/478052)

**Supplementary Figure. 1**


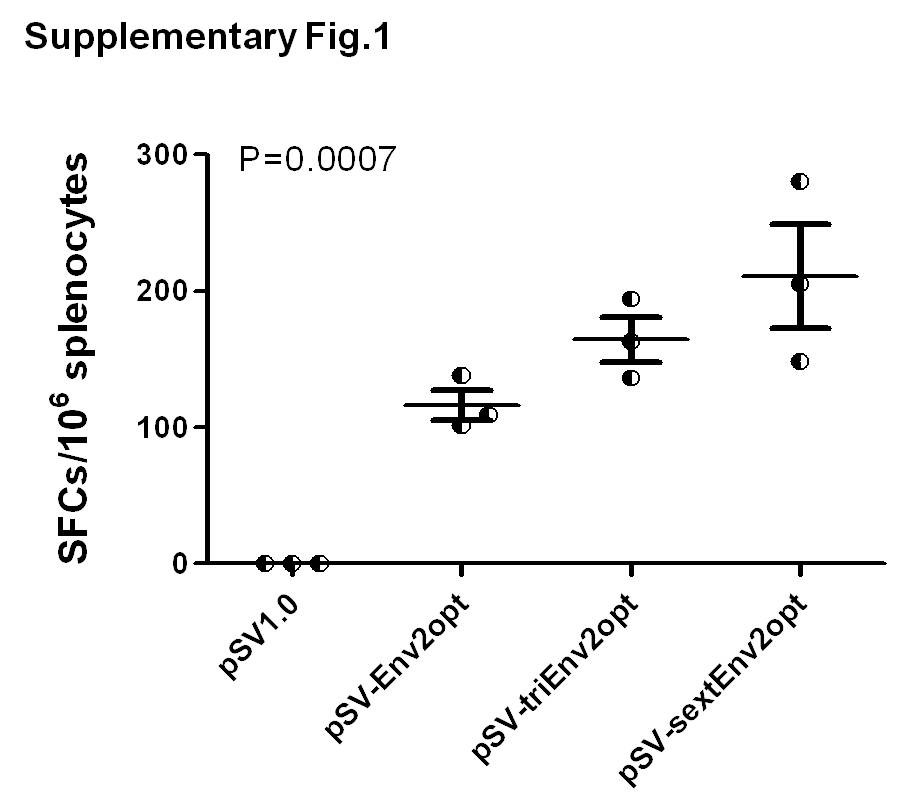

Supplement: Supplementary file 1 — To confirm the finding that increasing epitope copies could significantly augment the magnitude of specific T cells, 3 mice from each group were randomly selected for IFN-γ + ELISPOT assay. Our data showed that the frequency of IFN-γ + T cells could be significantly improved by increasing the epitope copies (P = 0.0007, one way ANOVA, supplementary figure 1). Additionally, a significant linear correlation was observed between the frequency of specific IFN-γ +CD8+ T cells and the epitope copy number (supplementary figure 2). [file 478052.f1.docx]

**Supplementary Figure. 2**


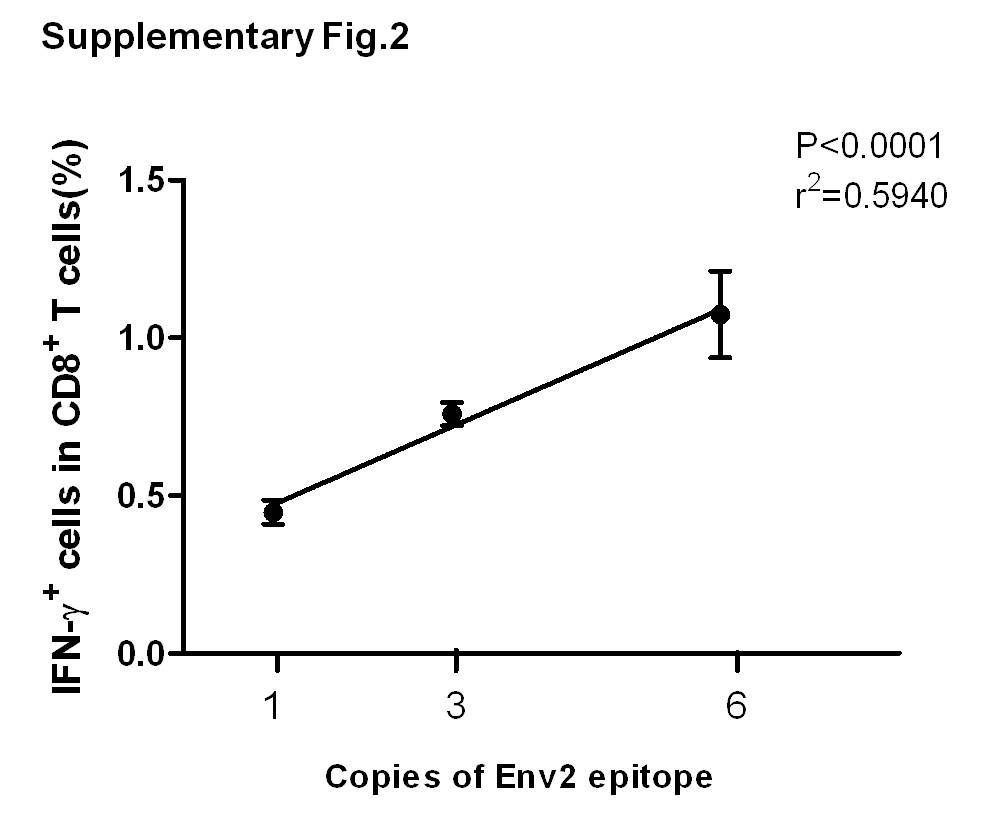

Supplement: Supplementary file 2 [file 478052.f2.docx]
